# Promoting Learning from Null or Negative Results in Prevention Science Trials

**DOI:** 10.1007/s11121-020-01140-4

**Published:** 2020-08-04

**Authors:** Nick Axford, Vashti Berry, Jenny Lloyd, Tim Hobbs, Katrina Wyatt

**Affiliations:** 1 NIHR ARC South West Peninsula (PenARC), University of Plymouth, Plymouth, UK; 2 NIHR ARC South West Peninsula (PenARC), University of Exeter, Exeter, UK; 3 University of Exeter, Exeter, UK; 4 Dartington Service Design Lab, Dartington, UK

**Keywords:** Randomized controlled trial, Null effect, Negative effect, Evaluation

## Abstract

There can be a tendency for investigators to disregard or explain away null or negative results in prevention science trials. Examples include not publicizing findings, conducting spurious subgroup analyses, or attributing the outcome post hoc to real or perceived weaknesses in trial design or intervention implementation. This is unhelpful for several reasons, not least that it skews the evidence base, contributes to research “waste”, undermines respect for science, and stifles creativity in intervention development. In this paper, we identify possible policy and practice responses when interventions have null (ineffective) or negative (harmful) results, and argue that these are influenced by: the *intervention* itself (e.g., stage of gestation, perceived importance); *trial design*, *conduct*, *and results* (e.g., pattern of null/negative effects, internal and external validity); *context* (e.g., wider evidence base, state of policy); and *individual perspectives and interests* (e.g., stake in the intervention). We advance several strategies to promote more informative null or negative effect trials and enable learning from such results, focusing on changes to culture, process, intervention design, trial design, and environment.

## Introduction

In his best-selling book, *Black Box Thinking*, Matthew Syed (2015) argues that aviation is much better than other fields in acknowledging and learning from performance failure. If an aeroplane crashes, the black box containing essential flight data is recovered, the data are analyzed, and any ensuing lessons are shared rapidly across the industry in order to improve engineering practice or pilot behavior and reduce the risk of a repeat event. He contrasts this with healthcare, contending that there can be a tendency to cover up or explain away treatment that is ineffective or harmful, or at least not to use this valuable information as an opportunity to learn and contribute to continuous improvement. We think there is a danger of similarly unhelpful behavior in prevention science when randomized controlled trials^1^ find a null or negative effect, and use this article to explore how to foster a more constructive approach. As will be seen, this might mean challenging the value of different types of research design in prevention science and what they can bring to improving the knowledge base from which learning can take place.

We recognize that there are complexities when trying to identify null or negative effect trials owing to issues with methodological quality and the pattern of results; taking the extremes, there is a world of difference between a well-conducted trial showing no effect on any measure of any outcome and a poorly executed trial showing no effect on the primary outcome but small effects on some measures of some secondary outcomes. The picture is further muddied by reporting practices that claim an effect when there is none. For the purposes of this article, we define null effect trials in terms of failure to disprove the null hypothesis on the primary outcome, despite what the authors may say or do, and negative effect trials as those that find a negative effect on the primary outcome.

Our interest in this subject was triggered by our experience of conducting several null effect superiority trials (Berry et al. 2016; Lloyd et al. 2018; Axford et al. 2020a, b, c). This prompted us to reflect on how we and other stakeholders responded, the relative value of the results (including whether they would even get published), and, in our (NA, TH) darker moments, whether the primary outcome meant a null effect was inevitable, whether the research design limited learning, and even whether the trials should have gone ahead in the first place. But our experience and concerns are not uncommon (Bonafide and Keren 2018; Oldehinkel 2018); a significant and possibly growing proportion of trials in prevention science and beyond (e.g., Kaplan and Irvin 2015) find no or even harmful effects.

Several explanations of this trend have been offered: (i) trials are conducted and reported more rigorously than previously owing to the advance publication of methods via online registries and protocols, the application of standards of evidence (e.g., Gottfredson et al. 2015), and the wide uptake by journals of reporting guidelines (Schulz et al. 2010); (ii) services as usual—the norm for control conditions—are improving, in part informed by results from earlier trials (the so-called “rising tide phenomenon”—Chen et al. 2016); (iii) an increasing number of trials are replication studies in new contexts that were not considered in the original intervention design and which, therefore, may not be favorable to finding positive effects; (iv) there are more independent trials with no involvement from program developers, who may have exerted deliberate or inadvertent influence on program delivery or trial methodology in the original studies and therefore inflated the effect (Eisner 2009; Gorman 2018); and (v) intervention developers and purveyors increasingly perceive a need to be endorsed by evidence-based program (EBP registries) in order to secure funding, which risks trialing programs prematurely (before establishing acceptability and feasibility).

In this context, it would be remiss if, as a field, we did not reflect on how to learn from well-conducted null and negative effect trials, particularly because how we respond affects not just what happens after a trial but how we think about and design interventions and tests of interventions. Yet widely used guidance on developing and evaluating complex interventions (Craig et al. 2008), and the draft update of that guidance which was distributed for consultation in 2019,^2^ make next to no direct reference to how to prepare for, consider and respond to null or negative results. Guidance *is* being developed on what to do next with effective interventions (Evans et al. 2019) but not, to our knowledge, what to do with those found to be ineffective. In short, there is a gap which this article seeks to help address.

In what follows, we describe how researchers often respond to null or negative trial results and the implications of their responses, set out what stakeholders might decide to do with the intervention following the results, hypothesize what influences those decisions, and finally propose a series of actions to promote learning from null or negative effect trial results. The suggested steps are designed to minimize the likelihood of unhelpful null effect trials—for example, those that are poorly designed or provide little or no explanation for the findings—and increase the proportion of trials which, even if they have null or negative effect findings, advance our learning. We draw on examples from our own and other people’s work in prevention science.

## Making Sense of the Trial Findings

There seem to be seven common investigator responses to null or negative effect trials in prevention science and beyond (Table 1). The evidence for some of these is compelling; for example, several extensive reviews covering a range of disciplines demonstrate publication bias (Hopewell et al. 2009; Duyx et al. 2017; Chow and Eckholm 2018). For others, such as forecasting delayed or sleeper effects, our observations are based on our own and others’ experience of reviewing programs for EBP registries (e.g., Martin et al. 2018).^3^ We readily acknowledge that the problems identified, such as failure to publish and conducting spurious subgroup analyses, are not unique to prevention science (Rosenthal 1979; Kasenda et al. 2014). Nor are we suggesting that researchers seek to be underhand. They (we) are part of a system and wider culture involving multiple players (e.g., developers, funders, policy-makers, commissioners, publishers, universities), so their (our) responses can be seen as rational acts in response to a complex set of incentives and constraints.

**Table 1:** Common researcher responses to finding null or negative effects in prevention science trials

Response	Effect	Legitimacy
1. * Don’t publish*: Researchers may not report null or negative findings, either because results papers are never submitted or because they are but scientific journals are not interested in publishing them (and the researcher gives up trying to get results into the public domain).	This contributes to a skewed impression of “what works” because the studies do not get picked up in systematic reviews and meta-analyses; specifically, evidence of effectiveness is likely to be exaggerated.	Failure to submit a results paper for publication is not necessarily a deliberate act, rather it can occur through inertia (although when an author is involved in intervention design or dissemination, this distinction becomes blurred). Journal editors and reviewers tend not to say that a lack of effect is the reason for rejection, but null effects rarely constitute the “ground-breaking” findings that journals invariably aspire to publish.
2. *Embark on fishing trips*: Researchers may embark on “fishing trips” to find evidence of impact. Usually this entails conducting spurious analyses in search of ad hoc subgroup effects.	The chances of finding false-positive results from a single dataset increase as more hypotheses are tested, so this practice can produce misleading results.	Moderator analyses specified a priori in the trial protocol or statistical analysis plan can be suitable, even if they are exploratory and acknowledged to be underpowered. However, there is widespread agreement that it is inappropriate to conduct ad hoc or theoretically uninformed moderator analyses in an attempt to find a positive effect for a subgroup.
3. *“Cherry pick” positive results*: In the context of predominantly null or negative results, researchers may single out any positive result, however small or practically insignificant, and accord it unwarranted prominence in the reporting of findings.	This creates the appearance of effectiveness, especially if the findings are “spun” in the write-up (e.g., by referring to “positive effects” in the abstract and relegating information about the lack of effect to the body text).	Given the difficulty of publishing null or negative findings, this response is unsurprising but problematic when it concerns any of the following: a secondary outcome or mediator; an interim data collection point; an outcome with marginal statistical significance (or the level of statistical acceptability is changed to make it “significant”); or a tiny effect that is unlikely to be of practical or clinical significance (even if it is *statistically* significant).
4. *Focus on methodological limitations*: Researchers may criticize measures or other aspects of trial design or conduct, implying that the test was unfair, or insufficiently rigorous, and that it therefore failed to uncover the “true” effectiveness of the intervention.	This casts doubt on the veracity of the findings (even when that is unfair), leading the reader to conclude that the intervention is potentially effective or of unknown effectiveness.	It is reasonable to identify limitations to trial methodology when reporting results, and for interested observers to critique the methods. Limitations in design or conduct might present a valid explanation for the lack of positive effects, with important implications for the interpretation of findings and conduct of future research. However, it is disingenuous to identify such problems only once results are known.
5. *Focus on poor implementation*: Researchers may attribute the null effect to a failure to implement the intervention with acceptable fidelity. To support the argument, extra analyses may be conducted to show that effects are observed when fidelity is stronger.	This suggests that the intervention would be effective if delivered as intended.	There is strong evidence for a positive association between fidelity and outcomes, so exploring this relationship is reasonable. However, care is needed not to use fidelity as an excuse once outcome results are known. Moreover, fidelity *x* outcome analyses should compare “compliers” in the intervention arm with a comparable group in the control arm (those who would have complied had they been offered the intervention) to avoid spurious positive associations.
6. *Focus on unsuitable context*: Researchers may contend that aspects of the context (e.g., organizational, cultural, political, economic) were unsuitable and help to explain why the intervention did not “work”.	This argument can be deployed to suggest that the intervention is effective but that it did not work *here*; put crudely, the problem is with the context not the intervention.	Contextual arguments may be legitimate, and can help with thinking about how to improve intervention development and implementation planning. However, they should not be used to cast doubt unfairly on null or negative effect findings, particularly if contextual issues were not considered before the findings were known.
7. *Forecast delayed or “sleeper” effects*: On failing to find effects at planned time points, researchers may argue that the study timeframe was too short and that positive effects will only become apparent in the future.	This argument can be used to imply that the intervention *is* effective but it was too soon to observe those positive effects.	Forecasting delayed effects may be reasonable if there are good theoretical or empirical grounds to justify it (e.g., observed effects on proposed mediators). When these are not present, it can cast doubt on null results unfairly, particularly in the absence of the means or intention of investigating longer-term effects.

It is important to acknowledge that the appropriateness of several of the behaviors identified is context-dependent, meaning that they can be acceptable, even desirable. For instance, if the trial quality does not meet the necessary standards of evidence such as those upheld by Blueprints for Positive Youth Development (Mihalic and Elliott 2015), it is appropriate that limitations to trial design or conduct cast doubt on the results. Similarly, context is dynamic and may change in unpredictable ways during the lengthy period of developing, piloting, and testing a complex intervention (Moore et al. 2019), such that it undermines intervention effectiveness. Moreover, forecasting delayed effects may be reasonable if there are good theoretical or empirical grounds to justify it (Hill et al. 2016)—for example, if effects were found for proposed mediators, or (non-significant) trends favored the intervention for aspects of development known to emerge more strongly as children mature. Since the impact of population-level interventions can take time to materialize, it is arguable that they should not be judged against traditional benchmarks of efficacy (Greenberg and Abenavoli 2017). Finally, attributing lack of effect to sample characteristics, say level of baseline difficulties, may be apposite if supported by exploratory moderator effects and the wider literature on the effectiveness of that category of intervention.

Nevertheless, both individually and collectively, *unhelpful* researcher responses to null or negative trial results limit learning. First, by unfairly casting doubt on robust findings, or artificially creating or inflating positive results, it contributes to a skewed impression of “what works” in a given subject area, inadvertently suggesting that some forms of intervention are more effective than they are (de Vries et al. 2018). This has the potential to cause harm. While there are techniques in meta-analysis to identify and compensate for publication bias (funnel plot, trim and fill algorithm, fail safe N), they are necessarily imperfect (Carter et al. 2019). Second, it contributes to research “waste”, which can increase risk and reduce benefits for service users. Accurate knowledge of earlier null or negative findings helps make future research more suitable and may even render some proposed studies unnecessary and irrelevant (Ioannidis et al. 2014). Third, it risks undermining the credibility of prevention science. Critics have highlighted what they perceive to be behaviors that artificially inflate reported intervention effectiveness (e.g., Gorman 2014); we should not ignore the issues. Fourth, it fosters a fear of null or negative results, which in turn stifles creativity and new approaches to intervention development and evaluation.

## Deciding What to Do with the Intervention

When a rigorous trial shows that an intervention is not effective, or that it is harmful, there are essentially three options for what to do with the intervention. Depending on the context, they may or may not represent appropriate learning.

The first possible response is to continue to commission or deliver the intervention. Stakeholders might accept the null or negative results but conclude that there are no better alternatives, or that the intervention is commendable for reasons besides its (non-)effect on outcomes. For example, despite the lack of effect in a trial of the PATHS social-emotional learning program in one city in the UK (Berry et al. 2016), the intervention continued to be commissioned in local schools for a further 3 years, at least in part because coaches, teachers, and students liked it. Of course, continuing to deliver the intervention may also happen if the results are not accepted by commissioners or are explained away by researchers.

A second response is to stop delivering and/or refining the intervention. This might take the form of decommissioning an established intervention or, if evidence accumulates from several null or negative effect trials of essentially similar programs albeit with different heritage or branding, de-implementing a class of interventions (Norton and Chambers 2020). Of course, if an intervention only existed as part of a trial, as in the school-based obesity prevention program tested in the Healthy Lifestyles Program (HeLP) trial (Lloyd et al. 2018), there may be nothing to decommission, but further development might cease. Additionally, when evidence from numerous null or negative effect trials accumulates, developers of health guidelines, such as the National Institute for Health and Care Excellence (NICE) in the UK, may issue “do not do” recommendations for clinical practices that should be discontinued or not used routinely.

A third response is to adapt the intervention and then test those changes. The rationale is that the trial results are broadly trustworthy and yield important lessons that need to be acted upon. In such cases, it is deemed premature to cease delivery but continuing with the intervention unchanged is not viable. In this way, the trial results are used as a platform for intentionally improving the intervention. Decisions about what to adjust are likely to be informed not only by outcome patterns but also, where available, by process evaluation results, not to mention wider evidence and expert opinion. Examples of this option include the reworking of a group parenting program (Ghate 2018) following a null effect trial (Simkiss et al. 2013) and the rapid cycle testing of adaptations to the Family Nurse Partnership home visiting program (FNP National Unit and Dartington Service Design Lab 2020) following disappointing trial results (Robling et al. 2016).

Such practice and policy decisions arise from a range of stakeholder responses which, we hypothesize, are shaped by the following four sets of potentially competing and interacting factors (Table 2). Exactly how these impact on decision-making is complex: their importance will vary by stakeholder and may change over time. We have derived these factors from our collective experience of responding to trials in which we have been directly involved as well as from our observations of other researchers and stakeholders.

**Table 2:** Influences on what happens to an intervention following a null or negative effect trial

Factor	Continued or future delivery of the intervention in its current form is *more* likely	Continued or future delivery of the intervention in its current form is *less* likely
*The intervention*		
Stage of gestation	Intervention is mature and widely commissioned	Intervention is new or early in its development
Perceived importance	Well established and politically important	Lower profile and limited political importance
Implementation feasibility/acceptability	Easy to deliver well, liked by practitioners/users	Hard to deliver well, disliked by practitioners/users
Outcome(s) targeted	Considered important (is a potential threat to health)	Considered less important (not a threat to health)
*Trial design, conduct, and results*		
Quality of trial design and conduct	Concerns about quality undermine confidence in results	Judged to be high quality and reliable
Pattern of outcome results	Somewhat inconsistent or inconclusive	Consistent and conclusive null/negative results
Context in which trial was conducted	Deemed significantly different to new/current context	Deemed to be similar to new/current context
Insight into reasons for the result	Explained by methodological or delivery issues	No reason to doubt or explain away the result
Nature of the control condition	Intervention of interest (I) vs. similar intervention (C)	Modification (I) vs. original intervention (C)
*Context for decision-making*		
Evidence base for intervention	Multiple other trials with positive effects	No other trials, or other evidence equivocal
Wider evidence base	Similar interventions not obviously superior	Similar interventions show positive effects
Policy and practice imperatives	Need to do something, and nothing clearly superior	Some discretion about acting, or superior alternatives
Political and economic situation	Limited resources, and “better” alternatives cost more	Resources allow more effective but costly alternative^a^
*Perspectives and interests*		
Investment in the intervention	Strong psychological or financial investment	Weaker investment, permitting more detached stance
Outlook on evidence (particularly trials)	Skeptical about evidence-based practice and/or trials	Sympathetic towards evidence-based practice/trials

^a^Only applies to existing interventions, not those delivered solely in the context of a trial

### The Intervention

An important issue is where the intervention is in its gestation. Finding a lack of effect early in its development is arguably less of an issue, and therefore easier to deal with, than if the intervention is considered to be mature and commissioned widely; the emphasis for newly developed interventions can be put on learning and re-design as there is little, if anything, to de-implement. Indeed, guidance on developing and evaluating complex interventions includes feasibility and piloting stage as a critical stage in the process (Craig et al. 2008).

A related factor concerns the profile and perceived importance of the intervention. If it is well established or politically important, for instance because it has been introduced by or received significant funding from government, it may be “too big to fail”, leading perhaps to a temptation to dismiss the results or plow on regardless with implementation and scale-up.

A further intervention-related factor is the degree to which it is possible to implement the intervention easily and well and whether it is acceptable to practitioners and users. An intervention that is well received or superior to its competitors in these respects may be more likely to continue to be commissioned, despite trial results showing no effect (see the PATHS example above).

Finally, the outcome(s) that the intervention seeks to address influences how trial results are treated. Specifically, some outcomes might be regarded as more important than others, for instance in terms of threat to health or cost to society if not achieved, such that null or negative results spur stakeholders into action in terms of discontinuing or modifying the intervention.

### Trial Design, Conduct, and Results

An assessment of the quality of the evaluation design and conduct likely has a bearing on stakeholders’ responses to null or negative trial results, since this affects whether the results need to be taken seriously—good *internal* validity—or instead should be viewed with caution. Such assessments may be conscious and well informed, as when trials are reviewed formally against standards of evidence for the purposes of populating online registries of EBPs, or arrived at rather more subliminally or casually—for example, based on the perceived caliber of research team members or the institutions they represent.

The pattern of the null or negative effects is also predicted to be an important factor: what proportion of outcomes are affected, and at what time points; are they primary and/or secondary; how important are any positive effects (even if few), whether theoretically (e.g., hypothesized mediators, knowledge vs. behavior) or in terms of the perceived veracity of the measure (e.g., independent observation vs. self-report); and what are the sizes of effects and how precise are they? It has been suggested that a trial is *informative* when it allows us to determine with confidence that an intervention is either effective or ineffective, and *uninformative* when—owing to the confidence interval being so wide (and precision so low)—it is consistent with the intervention being effective, ineffective, or harmful (Lortie-Forgues and Inglis 2019). If a clear picture emerges, suggesting little reason for optimism, it is likely to steer responses a different way (for example, towards discontinuation or modification) than if there is uncertainty or even a glimmer of hope (in which case protagonists might advocate conducting another, typically larger, trial).

Next, the context in which the trial was conducted and its similarity to the context in which results are to be interpreted and applied is likely to affect how different stakeholders respond. There has been a growing appreciation of the importance of the *external* validity of trials in recent years, such that questions are frequently raised about whether what works *there* will work *here* (Cartwright and Hardie 2012). Usually, debate centers on whether or not to import programs found to be effective in other countries, but equally we might ask how much weight to assign to a null effect trial in a different socioeconomic, political, cultural, or organizational context, or whether a null or negative effect in a “home” context counts for more than several positive “away” trials.

A further factor related to trial design that we hypothesize will affect how stakeholders respond to null or negative effects is the extent to which it generated insights that help to explain the results. Many—until recently, most—prevention trials focus on the effect on outcomes and pay less attention to process and mechanisms. This is changing (see below, also Moore et al. 2015), but arguably having a sense of *why* something was ineffective or harmful makes it easier to accept the result and learn from the findings.

Last is the nature of the control condition. Some trials compare a modified version of an intervention with the original (the control), or pitch the intervention of interest against a similar intervention (a so-called “head-to-head” trial). In the case of the former, the failure to add value to the original may make it easy to discontinue the modified version, while in the latter, a null effect may be interpreted positively (the intervention is not inferior) and lead to continuation of the intervention.

### Context for decision-making

One aspect of the context in which the trial results are reported is the evidence base. Whether the trial in question is the first of the intervention or the newest of several arguably has an effect on how stakeholders respond to the findings. Specifically, a null or negative result produced by the sole evaluation might be construed by some as a disaster, but the same result could be shrugged off if the trial is one of a series on the same program and earlier high-quality studies yielded overwhelmingly positive results. For instance, several EBPs with null effect trials in the UK, such as Functional Family Therapy (Humayun et al. 2017) and Multisystemic Therapy (Fonagy et al. 2018), nevertheless achieve the highest rating on the Early Intervention Foundation Guidebook^4^ owing to a preponderance of evidence in their favor from other studies. Then, there is the wider evidence base. If there are studies of similar interventions, their outcomes and the contexts in which they took place will shape the interpretation of disappointing findings. For example, a null effect might be taken as reason to discontinue an intervention if there is accumulating evidence that other approaches are more beneficial.

Another aspect of context relates to policy and practice imperatives. There may be a legal or moral obligation to do something to address the problem that the unsuccessful intervention seeks to tackle, or a lack of choice of other evidence-based approaches in the field. Decisions about what to do next with an intervention following a null or negative effect trial are clearly different in these circumstances compared with a situation where there is no obligation to intervene or a range of options from which to choose.

There is also the political and economic context. In a climate of public sector austerity, for instance, or faced with an external shock such as the Covid-19 pandemic, the need to respond to a problem quickly using the limited resources at one’s disposal usually takes precedence over careful consideration of the evidence. It would not be surprising, therefore, if an intervention shown in a trial to be ineffective continued to be implemented instead of more effective alternatives, especially if the latter cost more.

### Perspectives and Interests

The final set of factors revolves around the individuals concerned and, at a collective level, the organizations or interests they represent. Their perception of the aforementioned factors—the intervention, the study, and the context in which decisions are to be made—is shaped by their position, experiences, interests, beliefs, and predispositions. At the simplest level, program developers, funders, commissioners, practitioners, and evaluators all face different pressures and, as such, sometimes have competing priorities. It is difficult to disentangle how these play out, but we hypothesize that a critical factor will be how much individuals and the bodies they represent have invested in the intervention, whether financially, psychologically, organizationally, or politically. For example, practitioners are likely to have a stronger stake in a currently commissioned intervention than a new innovation developed by researchers; they may consider that an embedded and valued intervention has a legitimacy that should not be overridden by results from a trial. Some interventions are even synonymous with the organization that developed or delivers them, in which case a null or negative effect trial could have far-reaching repercussions at both structural and personal levels (such as loss of livelihood). It is much harder for individuals in those organizations to advocate discontinuing the intervention than it is for a dispassionate service commissioner needing to demonstrate value for money, or even an academic developer whose intervention exists primarily for research purposes.

The extent to which stakeholders endorse the value of trials as a robust means of generating evidence will also affect their response to null or negative effect trial results. A skeptic might not be overly concerned, preferring instead to prioritize other types of evidence or evaluation methods.

## Towards a More Constructive Approach

We recognize the interactive and dynamic nature of the factors outlined above, which makes it difficult to identify any single factor that explains how stakeholders respond to null or negative trial results. As researchers, we need to be sympathetic to and mindful of the conflict such results might create between and within stakeholders at many levels. For this reason, it is necessary to enable and support open and honest but potentially difficult conversations that take account of the wider context in which interventions are (or are not) implemented. Even so, some responses to null or negative trial results are arguably more constructive than others. So how do we cultivate a stronger culture of learning in response to evidence that an intervention was ineffective or harmful, and in so doing foster a climate for intervention design and testing that encourages learning *for the field* (i.e., beyond benefit for that specific intervention)?

In the Appendix, we set out a series of actions that can help to achieve this goal. Some pre-empt the problem by minimizing the likelihood of conducting null or negative effect trials. Others are concerned with preparing for such results so that learning is maximized should they materialize. The remainder focus on acknowledging and sharing null or negative effects and minimizing the temptation to manipulate or dismiss them. Collectively, they span the chronology of a trial from its inception through design and conduct to reporting; the right steps taken early on make it easier to act appropriately later. While some actions are arguably novel, several are advocated by others as part of best practice in developing and evaluating complex interventions (e.g., Craig et al. 2008; Davies 2013), in which case we seek to highlight their value in the current context. We would also argue that the actions are mutually reinforcing. For example, the process of considering results openly and honestly is more likely if efforts have been made to foster a collegiate learning culture. Broadly the actions identified fall into five categories.

### Culture

It is necessary to cultivate a *learning* culture among key stakeholders, that is, those people who will shape the decision about what to do with the intervention following the trial. This requires agreeing why the trial is being conducted, namely to learn about an intervention’s effectiveness and factors that contribute to this, with a view to improving the quality of services provided for children and families. The influence may be direct. For instance, provision may be enhanced by the incorporation of the intervention if it is found to be effective, or by efforts to improve the intervention if the results are equivocal or disappointing, or by replacing it with something that is more effective. Lessons from the evaluation may also contribute to services more indirectly through being picked up in systematic reviews or meta-analyses, which in turn have the potential to shape policy and practice. While achieving consensus among key stakeholders about trial purpose and value may be challenging, failure to do so will seriously undermine efforts to respond appropriately to the results should they be null or negative.

A learning culture can further be enhanced by managing expectations about results, namely the possibility of null or negative results (based on precedent), and by articulating likely and unlikely scenarios, such as the relatively common experience of seeing some effects on some measures of some outcomes and the rare experience of finding large effects on most outcomes. In order to reinforce a sense of openness and realism among stakeholders, it may help to develop outline plans for communicating positive, mixed, null, or negative results publicly. The overarching aim is to counter the erroneous belief that the trial will unquestionably prove the intervention to be effective and thereby give it a ticket to scale.

The aim should also be to encourage a *collegiate* culture, so that investigators and key stakeholders, especially program developers, feel that they are working together on a shared endeavor. This requires early and ongoing consultation, partly to understand different perspectives, motivations, and needs and thereby identify potential tensions but also to discuss trial design and conduct. For example, agreeing outcome constructs and measures before the trial commences guards against the temptation to criticize or regret the choice of measures post hoc once disappointing results are known and thereby undermine confidence in the null or negative effect. Failure to work together can create an adversarial culture in which, for instance, the deliverers of the intervention feel “done to” or under surveillance, which in turn contributes (unsurprisingly) to a reticence to accept and act on results.

### Process

In addition to working collaboratively, learning from null or negative results is more likely if the process of conducting the trial is done carefully and thoughtfully. There are various aspects to this. First, a definitive trial should only proceed if it is clearly necessary and appropriate, meaning that *all* of the following apply: (i) it has a plausible evidence-informed theory of change; (ii) potential harms have been considered and ruled out; (iii) intervention feasibility and acceptability have been established; (iv) there is genuine uncertainty about intervention effectiveness relative to the control (“equipoise”); (v) alternative methods of impact evaluation are unsuitable; and (vi) key stakeholders agree that a null or negative result is as worthy, interesting, and publication-worthy as a positive result. If an established or scaled intervention lacks a sound theory of change, efforts should be made to develop one retrospectively before proceeding to a trial, for example through an evaluability assessment (Davies 2013). Moreover, since many purportedly “innovative” interventions are highly derivative, it is arguable that testing their effectiveness in a definitive trial is unlikely to tell us anything important that we do not already know. In these cases, time and effort would be better spent improving the intervention so that it better embodies features known to be associated with or predictive of stronger effects. For example, a structured approach to doing this has been used to strengthen juvenile justice provision (Lipsey et al. 2010).

Second, conducting an internal or external pilot trial affords the opportunity to “fail” early, quickly, and insignificantly when the stakes are low and learn the lessons from this, so minimizing the likelihood of “failing” late, slowly, and at considerable cost and time in a definitive trial when the stakes are higher. For example, if a pilot trial indicates that the required sample size to detect a statistically significant effect in the main trial is too big for the planned recruiting sites to manage, this can be addressed by increasing the number of trial clusters or even abandoning the move to a definitive trial if cost and feasibility outweigh benefit (e.g., Segrott et al. 2015). Additionally, if the pilot uncovers problems with recruitment processes or the precision of the outcome measure, both of which could increase the probability of a null effect trial, then remedial action can be taken.

Third, if a definitive trial proceeds, it should be terminated early if appropriate. Developing and, if necessary, applying “early stopping rules” means that if it becomes apparent during the trial that there is likely to be a null or negative effect, for example owing to poor uptake or implementation problems, the study can be ended early, thereby minimizing research waste and potential harm to participants. In doing this, it is important that sequential analyses are conducted in order to avoid ending a trial prematurely based on incorrectly predicted futility and thereby inflating type I error rates.

Fourth, results need to be considered by members of the trial team and other stakeholders in a way that encourages dispassionate and thoughtful analysis. Specifically, process evaluation results should be shared first, allowing time for discussion about implementation fidelity in order to hypothesize why the intervention may or may not have worked and for whom, with outcome results only being shared second and, critically, without initially revealing the identity of the trial arms. To our knowledge, this is not common practice, but based on our own (VB) and colleagues’ recent experiences—in the E-SEE and Engager trials respectively (Bywater et al. 2018; Kirkpatrick et al. 2018)—we contend that it promotes less biased reflection on findings, and discourages the tendency to search for reasons to explain away disappointing outcome results.

Fifth, results need to be reported openly and fairly, in other words to accept them for what they are and share them with others. Minimizing the temptation to manipulate or dismiss results in the ways described above starts by setting parameters early in the process. Thus, we should state success criteria before the trial commences, register the trial, publish the protocol, and put in the public domain an analysis plan that aligns with the protocol. This increases accountability by limiting the opportunity to bury undesirable findings or give undue weight to effects on secondary outcomes or for subgroups. Critically, the results need to be published, and in line with best practice (Grant et al. 2018). If it proves impossible to get the paper accepted in a high-impact journal, options include submitting it to a journal that operates “results-free” peer review, meaning that acceptance is based on methodological quality rather than findings, or one specializing in null result studies (e.g., *Journal of Articles in Support of the Null Hypothesis*). Other repositories include PsychFileDrawer.org, which focuses on “serious replication attempts in all research areas of psychology—whether they succeeded or failed” and encourages online discussion of findings.

### Intervention Design

Much has been written about good intervention design elsewhere (for a review, see O’Cathain et al. 2019), so here we highlight only a few points. One is the importance of drawing on relevant literature that has been appraised carefully and is deemed to be reliable. This, in turn, requires that the quality of basic research is improved, for instance through study pre-registration, better data sharing, and more replication research (Lortie-Forgues and Inglis 2019). Next, design is likely to be further strengthened by building trusting relationships with intervention developers, professional development providers, and people with lived experience of the issue targeted by the intervention and collaborating with them in a process of human-centered co-design (Lyon and Koerner 2016). A further consideration should be intervention context, specifically the factors (e.g., political, organizational, cultural, social, economic, geographical, financial) that are anticipated to impact on implementation and therefore outcomes. An implementation research framework (e.g., Damschroder et al. 2009) and guidance on how to take account of context in intervention research (Craig et al. 2018) could usefully inform this exercise, shaping both intervention design and implementation strategy. Lastly, possible unintended adverse effects of the intervention (which may contribute to null or negative effects) should be considered and the design adjusted accordingly (Bonell et al. 2015). In addition to asking stakeholders to consider likely adverse effects freely and without prompting, it can be useful to work together through common types such as psychological stress, widening health inequalities, deviancy training, and opportunity costs (Lorenc and Oliver 2013).

### Trial Design

Trial design has a significant bearing on the extent to which the results are conducive to learning. Several steps can be taken to minimize the likelihood of results leaving ambiguities in the event of null or negative effects, thereby making them more informative. Equally, certain actions enable the exploration and therefore potential elimination of competing explanations for an intervention being ineffective or harmful, thereby pointing to possible improvements or practices to avoid.

The first is ensuring that the study is adequately powered, either by increasing sample size if practical or, if not, by focusing on more targeted subgroups or using more targeted outcome measures (Lortie-Forgues and Inglis 2019). This helps to avoid finding no effect because the sample was too small. Second, it pays to record carefully the services received by control arm participants. If they significantly exceed those received by intervention participants, or resemble the intervention, it may help to account for null or negative effects. Third, the timing of follow-up points should be calibrated according to theoretical and empirical evidence on when outcomes are likely to be observed. If an effect on the primary outcome is not expected until 12 months post-intervention, this data collection point should be in the study design. Fourth, statistical mediation analysis (O’Rourke and MacKinnon 2018) and qualitative techniques such as contribution analysis (Mayne 2008) can be used to explore whether the theory of change has materialized in practice, which may help explain null or negative effects. Fifth, all aspects of fidelity need to be recorded, including delivery (dose, adherence, quality, responsiveness), implementer training, and the degree to which participants enact what the intervention focuses on (Borrelli 2011). This helps with determining if and how poor fidelity accounts for a lack of effect. Sixth, there is much value in conducting pre-specified ancillary analyses that explore the relationship between outcomes on the one hand and sample characteristics and fidelity on the other. This involves sufficiently powered subgroup analyses to explore whether some types of participant benefit more than others, and complier average causal effect (CACE) analysis, which compares “compliers” in the intervention arm with a comparable group in the control arm (Hewitt et al. 2006). Finally, robust data should be gathered on implementation context, as this affects intervention effectiveness (Craig et al. 2018), and possible adverse or neutralizing effects (see above). Many of the suggested actions here align with the trend towards mixed methods and realist trials (Hesse-Biber 2012; Bonell et al. 2012), which move from answering “Does it work?” to “For whom does it work, why and in what context?”

### Environment

As indicated earlier, the behavior of investigators and key stakeholders is shaped by multiple incentives and constraints. For this reason, their ability to enact our recommendations demands a suitable infrastructure and supporting climate. This requires collaboration from a number of actors besides investigators and program developers (the audience for most of the preceding recommendations).

First, funders need to be willing to pay for feasibility studies and pilot trials, and for “thicker” trials that incorporate robust process evaluations and analyses of mediators, moderators, and fidelity *x* outcome interaction effects. They should also fund—and indeed insist on—protocol sharing and publication of results, regardless of what form they take. If investment in trials is seen as part of a developmental process, there is also a case for a guaranteed “improvement fund” should re-design be the preferred option or a protected “decommission fund” if an established intervention is deemed to have no future. While these suggestions have cost implications, funders can save money by being more selective about the trials they fund, which might include paying for evaluations that use other methods where suitable.

Second, publishers—supported by journal editors and editorial boards—need to make it easier to publish null and negative trial results. Strategies might include results-free peer review or accepting results papers “in principle” on acceptance of a protocol article. Additional steps to support honest reporting of results and reduce potentially biased post hoc critique of methods include only publishing trial results if the protocol and analysis plan are in the public domain, making more space available in journals for trial protocols, and allowing room in journals for authors and critics to debate the merits of a given trial design *before* results are known (Chan and Hróbjartsson 2018).

Third, intermediary organizations concerned with promoting research utilization could play a valuable role in supporting developers and purveyors with intervention design, improvement, and evaluation. This includes helping them to develop interventions that are less likely to produce null or negative effects, which might entail assistance with finding and applying existing research evidence in the context of a human-centered co-design process. It might also involve adapting interventions sensibly in the light of disappointing findings, or encouraging the use of evaluation methods that contribute to intervention improvement rather than progressing prematurely to a trial.

Fourth, EBP registries should encourage the appropriate generation and use of evidence. This might entail providing credit for robust evidence of a null or negative effect and issuing guidance on how to weigh such evidence, for example highlighting that depending on other factors (see above) it need not mean discontinuing the intervention. It could also involve providing stronger ratings for well-conducted non-trial impact evaluations that nevertheless go some way towards attributing causal inference, and highlighting programs that display features or common elements of effective interventions (even if they have not themselves been evaluated experimentally). These steps would mitigate the pressure felt by developers and purveyors to subject their intervention to a trial prematurely in order to attain a rating that will, they believe, increase its likelihood of being commissioned.

Lastly, academic institutions could credit investigators who share trial protocols (Chan and Hróbjartsson 2018) and publish null or negative trial results.

## Conclusion

We have sought to recast null or negative trial results as something to learn from, not fear. The learning should be for the field and not restricted to the intervention in question. This depends on trials being designed and conducted with a learning mindset and in a commissioning and policy climate that encourages innovation and experimentation and reduces associated disincentives. There is also a need for researchers, funders, and developers to reflect on the fact that while simple behavioral interventions are easier to implement and to evaluate through trials, they are less likely to work in tackling complex social and health problems with complex causes (Ghate 2016; Rutter et al. 2017). In other words, the system that encourages such activity inadvertently increases the likelihood of null effect trials.

More empirical research is needed into how stakeholders manage and respond to null and negative effect trials and the factors that predict this, since this will help with understanding the barriers to and facilitators of learning. This should entail a combination of desk-based research to code responses to null or negative effect trials and in-depth interviews with key stakeholders about post-trial decision-making to illuminate what happened and why. We also plan to conduct a Delphi exercise to synthesize multiple stakeholders’ perspectives on our recommendations with a view to producing guidance for investigators. In the meantime, we look forward to a time when there will be fewer but more informative null and negative effect trials—essentially more mixed method trials of potentially ground-breaking innovations—and a stronger emphasis on applying the lessons from such studies to embedded practice.

## Appendix. Recommended actions to promote learning from null and negative effect trials in prevention science^5^

**A. Culture**

Foster a *learning* culture among key stakeholders by:

- [1] agreeing pre-trial that the goal is to help improve population health outcomes, whether through the selected or other interventions, and learning how best to do this
- [2] agreeing the opportunities for learning (i.e., questions the trial will help answer)
- [3] managing expectations about outcomes (e.g., possibility of null or negative results)
- [4] planning for how to interpret and communicate results, whatever form they take

Foster a *collegiate* culture by:

- [5] engaging from the outset in regular and ongoing consultation about decisions regarding the intervention and trial

**B. Process**

Proceed carefully, thoughtfully, and collaboratively, so that:

- [6] a definitive trial is only conducted if necessary and appropriate, by:
  - [i] developing a clear and logical theory of change
  - [ii] considering potential harms and either putting in place mitigating actions or redesigning the intervention to reduce or eliminate potential harms
  - [iii] establishing intervention feasibility and acceptability
  - [iv] ensuring that there is genuine uncertainty about intervention effectiveness relative to the control (“equipoise”)
  - [v] obtaining consensus among key stakeholders that a null or negative result is as interesting, useful, and publication-worthy as a positive result
  - [vi] considering and ruling out alternative (non-trial) methods of impact evaluation
- [7] the trial is terminated early if appropriate, by developing and, if necessary, applying early stopping rules
- [8] results are considered in an honest way, by sharing process evaluation results within the research team first, then sharing the outcome results (blind to trial arm in the first instance)
- [9] results are reported openly and fairly, by:
  - [i] stating success criteria before the trial commences, in particular the primary outcome(s) and minimum effect size that is of practical significance
  - [ii] registering the trial on a relevant online database, publishing the trial protocol, and developing (and making publicly available) a detailed analysis plan (statistical and qualitative) that aligns with the protocol
  - [iii] publishing the results as fully and in as publicly accessible a way as possible

**C. Intervention design**

Design the intervention in such a way that it is less likely to have a null or negative effect, more likely to be suitable for the context and more likely to be implemented well, by:

- [10] drawing on literature that has been appraised as being reliable to inform the intervention design
- [11] co-designing the intervention with practitioners, professional development providers, and people with lived experience of the issue
- [12] identifying at the outset possible unintended adverse effects that might contribute to null or negative effects, and either redesigning the intervention completely or making adaptations accordingly
- [13] understanding and taking account of context and the system in which the intervention will be implemented

**D. Trial design**

Design and conduct the trial in such a way that it is:

- [14] less likely to leave ambiguities and more likely to be informative, by:
  - [i] ensuring the study is adequately powered
  - [ii] recording what intervention and control group participants are receiving by way of the intervention and other (non-intervention) services
  - [iii] calibrating follow-up data collection time points based on theory and empirical evidence on when effects are expected to be observed
  - [iv] gathering robust data on all aspects of fidelity
  - [v] exploring mechanisms of impact both qualitatively and quantitatively
  - [vi] undertaking pre-specified and sufficiently powered moderator analyses
  - [vii] undertaking appropriate fidelity *x* outcome analyses
  - [viii] gathering robust data on implementation context
- [15] less open to post hoc criticism, by agreeing measures and other aspects of design a priori (see above)
- [16] alert to possible adverse effects or at least neutralizing influences, by gathering appropriate data on such influences and undertaking relevant analyses

**E. Environment**

Enable all of the above by cultivating an infrastructure and climate that incentivize desired behaviors and disincentivize undesired behaviors on the part of investigators and program developers. This involves the following:

- [17] funders paying for: feasibility and pilot studies; “thicker” trials with substantial process evaluations and ancillary analyses; protocol sharing; open access results publication; alternative evaluation methods where suitable; post-trial action plans
- [18] academic publishers: mandating protocol publication prior to trial results publication; making more space for protocol sharing and debate on trial methods as specified in protocols; making space for publication of statistical analysis plans; offering results-free peer review; and accepting trial results articles “in principle” at the point of accepting a protocol for publication
- [19] intermediary organizations: providing support and training with intervention design/adaptation; and assisting developers and purveyors with service improvement and evaluation
- [20] registries of EBPs providing credit for: interventions subjected to a high-quality null effect trial; non-trial impact evaluation; and non-trialed programs assessed as displaying key features of effective programs
- [21] academic institutions crediting investigators who share trial protocols and publish null or negative trial results

## Footnotes

1 In this article, we refer primarily to superiority trials investigating the hypothesized added value of an innovation/intervention over a services as usual comparator. But the arguments may also apply to null results from equivalence or non-inferiority trials, which suggest that the new intervention is likely to be inferior to or not as good (by a defined margin) as standard practice or an alternative treatment.

2 https://www.gla.ac.uk/researchinstitutes/healthwellbeing/news/hawkeye2018onwards/march2019/headline_641840_en.html

3 Our experience has been with: Blueprints for Healthy Youth Development; the Early Intervention Foundation Guidebook; Project Oracle; the EMCDDA XChange database; Evidence2Success; and Investing in Children.

4 The Guidebook is a UK-based registry of prevention and early intervention programs: https://guidebook.eif.org.uk
